# γ-Propoxy-Sulfo-Lichenan Induces In Vitro Cell Differentiation of Human Keratinocytes

**DOI:** 10.3390/molecules24030574

**Published:** 2019-02-05

**Authors:** Stefan Esch, Maren Gottesmann, Andreas Hensel

**Affiliations:** University of Münster, Institute of Pharmaceutical Biology and Phytochemistry, Corrensstrasse 48, D-48149 Münster, Germany; s_esch04@uni-muenster.de (S.E.); m_gott05@uni-muenster.de (M.G.)

**Keywords:** lichenan, γ-propoxy-sulfo-lichenan, keratinocytes, differentiation, involucrin, keratin

## Abstract

Background: As non-cellulosic β-d-glucans are known to exert wound-healing activity by triggering keratinocytes into cellular differentiation, the functionality of a semisynthetic lichenan-based polysaccharide on skin cell physiology was investigated. Methods: γ-Propoxy-sulfo-lichenan (γ-PSL, molecular weight 52 kDa, β-1,3/1,4-*p-d*-Glucose, degree of substitution 0.7) was prepared from lichenan. Differentiation of primary human keratinocytes was assayed by the protein analysis of differentiation specific markers and by gene expression analysis (qPCR). The gene array gave insight into the cell signaling induced by the polysaccharide. Results: γ-PSL (1 to 100 μg/mL) triggered keratinocytes, in a concentration-dependent manner, into the terminal differentiation, as shown by the increased protein expression of cytokeratin 1 (KRT1). Time-dependent gene expression analysis proved differentiation-inducing effects, indicating strong and fast KRT1 gene expression, while KRT10 expression showed a maximum after 12 to 24 h, followed by downregulation to the basal level. Involucrin gene expression was only changed to a minor extent, which was similar to loricrin and transglutaminase. Gene array indicated the influence of γ-PSL on MAP kinase and TGF-β mediated signaling towards keratinocyte differentiation. Conclusion: The propoxylated lichenan may improve wound healing by topical application to promote the terminal barrier formation of keratinocytes.

## 1. Introduction

The clinical treatment of wounds still has an enormous impact on the healthcare economy. Especially, skin ulcera causes problems in clinical practice and in significant parts of healthcare resources in nearly all countries worldwide, and are used for optimized wound healing [1]. While, in developing countries, acute wounds are mostly treated routinely by antiseptic and surgical procedures, most physicians and healers in developing and emerging countries depend on the topical treatment of acute wounds by using antibacterial agents, anti-inflammatory drugs, or entities with cell proliferation-inducing activity.

On the other hand, the treatment of chronic wounds still causes problems, with a high-impact, all over the world, e.g., leg ulcerations occur in about 1% of the population. In the U.S., chronic wounds affect 3–6 million patients with an estimated annual cost of $3 billion each year and a loss of over two million workdays per year [1]. The need for the development of innovative drug compounds with improved pharmacological activity for effective and evidence-based wound treatment is obvious.

Wound healing is a highly complex machine with a multitude of precisely regulated changes in the cell physiology of keratinocytes and dermal fibroblasts. The key players in the formation of epidermal tissue are the keratinocytes, which initially undergo strong proliferation, which is subsequently followed by cellular differentiation towards specialized barrier cells. Additionally, dermal fibroblasts, embedded into a complex extracellular matrix, which again influence the cell physiology of fibroblasts as well as the keratinocytes, will build up the connective tissue of the dermis. 

Loricrin (LOR), formed at the end of the differentiation process in the stratum granulosum, accounts for up to 85% of the differentiation-specific proteins in the cornified envelope and can form a strong protein network by the formation of transglutaminase-catalyzed interprotein linkages [2]. Major differentiation-specific proteins for the formation of the barrier function of keratinocytes are keratins (KRT, also called cytokeratins). During the differentiation process of keratinocytes, significant changes in the subtypes of keratins are observed, while undifferentiated keratinocytes in the stratum basale are dominated by KRT5 and KRT14, differentiation-specific cytokeratins KRT1 and KRT10 are found during the initial and late phases of differentiation [3]. This leads to typical cytological changes from desmosomes to the typical corneodesmosomes. Subsequently, filaggrin (FLG) reorganizes the KRT1 and KRT10 networks, which forms the typically flat-typed corneocytes. Transglutaminases (TGM) are Ca^2+^-dependent enzymes, linking the structural proteins of the cornified envelope to each other [4]. While TGM1 and 5 are mainly involved in the early phase of differentiation, TGM3 initiates late cellular differentiation [3]. In addition, more lipohilic molecules (e.g., lipids, cholesterol, and ceramides) are key players in the establishment of the final skin barrier. The precise regulation of the differentiation process is strictly regulated by different signaling pathways, which are controlled, among other things, by Ca^2+^, cell–cell contacts, and transforming growth factor TGFβ. 

Within the last years, systematic investigations on the potential of medicinal plants and natural products for improved wound healing have identified some plant-derived extracts and characterized natural products with stimulating activity on dermal fibroblasts and keratinocytes; these extracts and compounds can cause an increased tissue regeneration by stimulating the proliferation of dermal fibroblasts and keratinocytes [5,6,7]. 

Interestingly, only very few compounds have been described as interacting with the terminal phase of wound healing. This late phase is characterized by the formation of a correctly closed epidermal barrier, which on the molecular level is due to the cellular differentiation of keratinocytes [8,9,10]. This barrier function is closely related to the formation of the cornified envelope as a typical hydrophobic barrier on the cell surface of keratinocytes in the stratum spinosum. Typical marker proteins for the cornified envelope are KRT 1/10, involucrin (IVL), LOR, FLG, and TGM [3].

Despite the fact that many compounds are known to induce keratinocyte (and fibroblast) proliferation, a lack of compounds stimulating keratinocyte differentiation is obvious. Currently, only retionoids are clinically used for this reason, but as the risk-benefit ratio for these drugs is not favorable, their use is limited. Preclinical studies indicated differentiation-inducing effects of different β-d-linked glucans. For example, xyloglucan from *Tropaeolum majus* seeds, characterized by a β-d-1,4-glucose backbone and strong 1,6-branching, was shown to be a strong inductor of keratinocyte terminal differentiation by the inhibition of the activation of the epidermal growth factor receptor (EGFR) [8]. Other β-d-glucans such as the β-1,3/1,4-linked lichenan from *Cetraria islandica* and oligomeric chitosan strongly induce cellular differentiation [9,11]. From these data, it can be concluded that gel-forming polysaccharides with a β-glucan structure might be polymers that have a high-impact on wound-healing by influencing terminal differentiation, and therefore, stimulating an improved wound closure and barrier formation. 

Many β-glucans have limited solubility in water, and therefore, the practical use for the manufacturing of galenical formulations and wound dressings is often impaired. On the other hand, screenings of different β-glucans indicated that cellulose-like, unbranched β-1,4-d-glucans do not influence keratinocyte differentiation. Therefore, soluble cellulose derivatives (e.g., alkyl-substituted cellulose, carboxymethyl cellulose, etc.) cannot be used. Mixed-linked 1,3/1,4 β-d-glucans (e.g., lichenan) stimulate cell differentiation, but can have some negative physicochemical activities. At this point, a water-soluble lichenan derivative (e.g., propoxy-sulfo-lichenan) has been reported in the literature, which has been developed as an anticancer polysaccharide, strongly stimulating the innate immune defense [12]. This polysaccharide has been shown in the following study to exert differentiation-stimulating activity under in vitro conditions.

## 2. Results and Discussion

**Figure 1:**
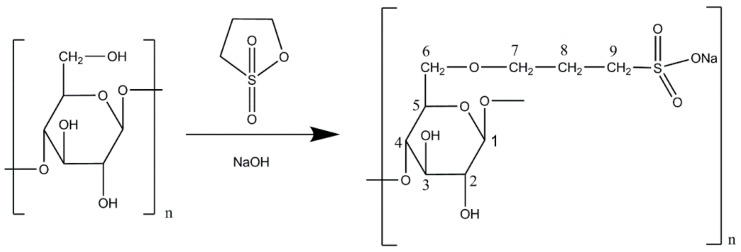
Synthesis of γ-propoxy-sulfo-lichenan (PSL) from lichenan and γ-propan sulton under alkaline conditions.

γ-Propoxy-sulfo-lichenan (γ-PSL, Figure 1) was prepared by the reaction of lichenan from *Cetraria islandica (*molecular weight 275 kDa, β-1,3/1,4-d-*g*lucopyranose, and ratio 1:3) with γ-propansulton under strongly alkaline conditions in a yield of 47% (*w*/*w*). In contrast to lichenan, γ-PSL had good cold-water solubility up to 25 mg/mL; higher concentrations up to 50 mg/mL resulted in gel formation. The mean molecular weight was determined by gel permeation chromatography with 52 kDa (Supplementary Data, Figure S1).

**Figure 2:**
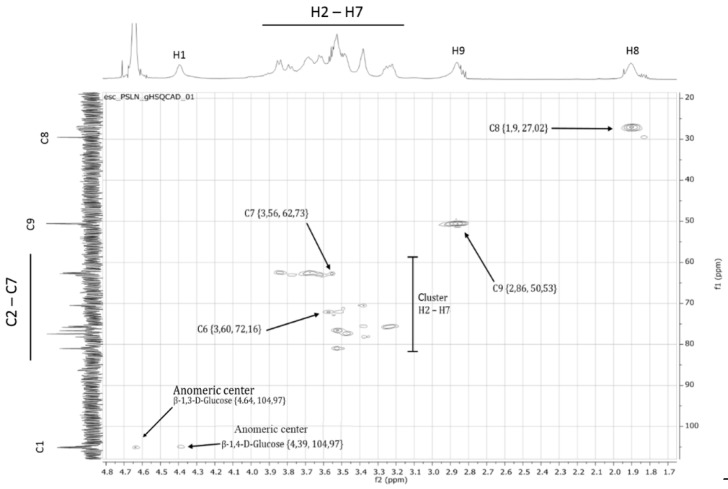
Relevant details of the ^1^H-^13^C-HSQC-NMR spectrum of γ-PSL. Additional signals of γ-PSL, in comparison to unsubstituted lichenan, are marked by arrows.

^1^H-NMR and ^13^C-NMR indicated the substitution of the polysaccharide; ^1^H-^13^C-HSQC-NMR (Figure 2) correlated the propoxy-sulfo side chains to the respective substitution at the glucose backbone. The signals of C8 {1.9, 27.02 ppm} and C9 {2.86, 50.53 ppm}, as well as those of the anomeric center {4.39, 104.97 ppm}, can be assigned clearly. Signals from C7 of the γ-propoxy-sulfo side chain {3.56, 62.73 ppm} can be related to the signal of C6 of the sugar moiety. The ^13^C-NMR C6 signal is typically shifted by about 10 ppm in the case of substitution with the sulfo-alkylated side chain {3.60, 72.16 ppm}. Additionally, the ratio of β-C1_1→3_ to β-C1_1→4_ can be calculated at about 1:2. As the ratio of 1,3/1,4-linkages in the unsubstituted lichenan has been determined as 1:3 (data not shown), this indicates that, during the synthetic derivatization of lichenan to γ-PSL, the glucan backbone gets partially cleaved at β-1,4 linked glucose residues. 

To investigate the influence of γ-PSL on the cell physiology of human keratinocytes, natural human epidermal keratinocytes (NHEK) were incubated with γ-PSL (100 μg μg/mL) and cell proliferation was determined by BrdU-incorporation ELISA [13]. No changes in the cell proliferation rates were observed (Supplementary Data, Figure S2B). Additionally, the influence of γ-PSL on cell viability was investigated in NHEK, using the quantification of the activity of mitochondrial succinate dehydrogenase by MTT assay over 48 h [14]. No signs of reduced cellular vitality were observed up to 256 µg/mL (Supplementary Data, Figure S2A). 

**Figure 3:**
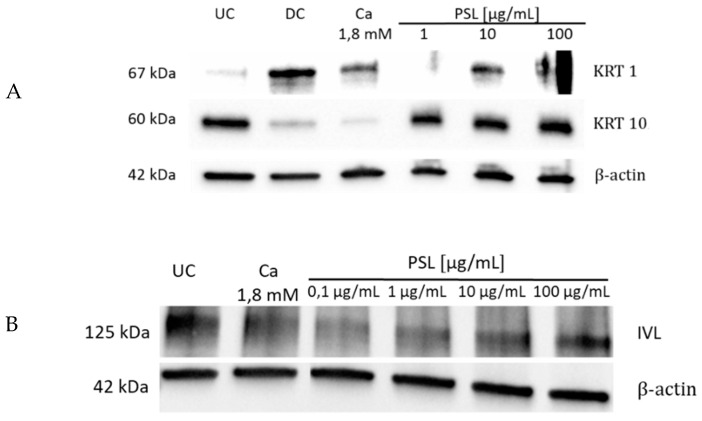
Influence of γ-PSL (1 to 100 μg/mL) on the expression of differentiation-specific marker proteins KRT1, KRT10 (**A**), and IVL (**B**) in natural human epidermal keratinocytes (NHEK) after 7 days of incubation. Loading control: β-actin (10 µg protein/sample); positive controls: Ca^2+^ 1.8 mM; DC: Double cell density, contact inhibition; and UC: Untreated control. Note: KRT1 antibody partly produced artifacts.

Regarding the influence of γ-PSL on cellular differentiation, NHEKs were incubated for 7 days with the glucan and the expression of the differentiation-specific marker proteins KRT1, KRT10, and IVL were monitored by Western blot (Figure 3). Ca^2+^-treated cell cultures were used as a positive control [15], acting on the calcium sensitive receptor and on the cell-cell adhesion, which can both trigger the keratinocytes into terminal differentiation. As it is a widespread problem, calcium-induced differentiation is, in many cases, not very reproducible in standard cell cultures; additionally, cell groups with increased cell density were investigated as positive controls, which can trigger differentiation of the cells via E-caderin [16].

γ-PSL clearly upregulated differentiation-specific KRT1, while KRT10 was only influenced to a little extent. γ-PSL at 100 μg/mL did not change IVL formation, while, interestingly, lower concentrations of the polymer lead to decreased IVL formation (Figure 3B).

**Figure 4:**
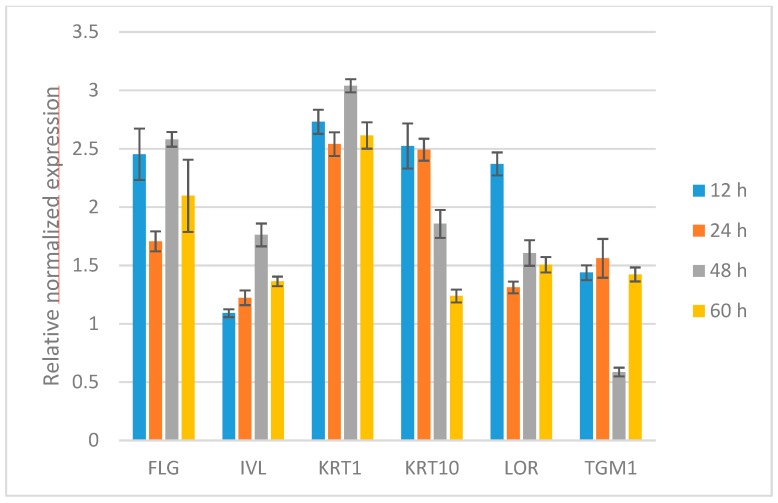
Influence of γ-PSL (100 µg/mL) on the relative, normalized gene expression of differentiation specific marker genes in NHEKs by time dependent qPCR over 12–60 h. FLG: filaggrin, IVL: Involucrin, KRT1: Cytokeratin 1, KRT10: Cytokeratin 10, LOR: Loricrin, and TGM1: Transglutaminase 1. Relative, normalized expression, related to the untreated control (expression = 1).

To prove the differentiation-inducing effect of γ-PSL on the gene level, time-dependent gene expression analysis by qPCR was performed in NHEKs over 12, 24, 48, and 60 h by monitoring the differentiation-specific early proteins KRT1, KRT10, and IVL, and the late marker proteins LOR and FLG as well as TGM1, responsible for the correct formation of the cornified envelope. Figure 4 displays the relative normalized expression of the respective marker genes at different time points, which are related to untreated control groups.

The respective gene expression data confirmed the findings from the protein assays, that is, a strong and fast upregulation of KRT1 and KRT10 is induced by γ-PSL, followed by a plateau-like constant gene expression of KRT1 over 60 h. In contrast, KRT10 gene expression showed a maximum after 12–24 h, followed by a reversion to the basal expression level. This can explain the differences between KRT1 and KRT10 on the protein level found within the protein analysis (Figure 3). In analogy to the data from protein analysis, IVL gene expression was also only changed to a minor extent (max. fold change 1.7). Interestingly, FLGs also showed a strong gene expression over 12–60 h, which is similar and comparable to that of the cytokeratines. Similar to the strong FLG expression at early stages, LOR also gets upregulated after 12 h (about 2.5 fold), followed by a lower gene expression at later stages. TGM gene expression was not stimulated to a higher extent.

**Figure 5:**
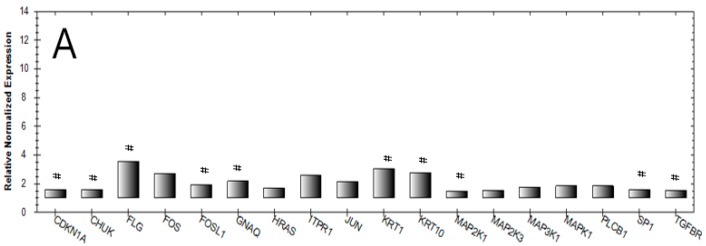

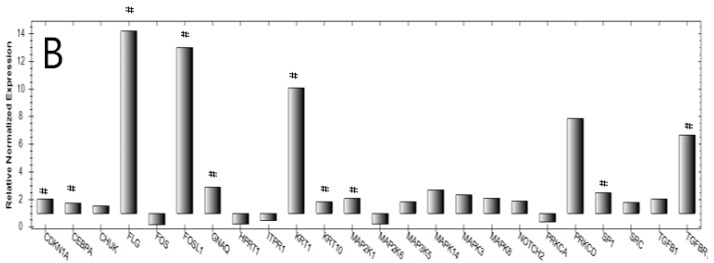
Quantitative Real-Time Array (Development-Keratinocyte differentiation H96 array—BioRad) after 60 h incubation of NHEK with γ-PSL (100 µg/mL) on the relative, normalized gene expression of differentiation specific genes in relation to the untreated control (normalized expression = 1); #: genes selected for subsequent qPCR cross validation. Data are related to the independent array experiments (**A**,**B**).

To investigate the γ-PSL inducing effect on keratinocyte differentiation and to gain deeper information on the influence of the glucan on the skin cell physiology and cell signaling, a gene array by the use of NHEK was performed, which mainly covered genes that are involved in cellular differentiation. For quantitative evaluation, a fold change of gene expression of <0.5 resp. >1.5 was related to the untreated control and was specified (Figure 5). Interestingly, 10 genes were identified, for which an influence of γ-PSL (100 µg/mL) can be assumed (Table 1). 

The results from this gene array are, in principle, congruent with the data obtained from the protein analysis and the gene expression study: KRT1 and KRT10 gene expression is clearly upregulated. Additionally, gene expression of the late differentiation marker FLG is strongly increased. Interestingly, typical signal cascades are also stimulated by γ-PSL; activation of cyclin-dependent kinase inhibitor 1 (CDKN1Ap21) induces cell cycle arrest, which subsequently leads to the switch of the cell into the G0 phase [17]. Again, this process is known to initiate cell differentiation in an irreversible way. 

Upregulation of Fos-related antigen 1 (FOSL1), a part of the AP1-transcription factor complex, as well as the transcription factor Sp1, indicated that gene regulation in the cell after contact with γ-PSL has clearly shifted towards cellular differentiation. AP1 and Sp1 are known to activate in the promotor regions of the transcription of the differentiation markers LOR and IVL [20]. In addition, upregulation of the receptor of TGFB1 indicates a shift in cell physiology towards terminal differentiation. From these data, it can be concluded that the polysaccharide γ-PSL acts on the surface of skin cells, initiating an intracellular MAP-kinase signaling, which activates TGFβ-mediated cell signaling towards the induction of the cellular differentiation. 

**Figure 6:**
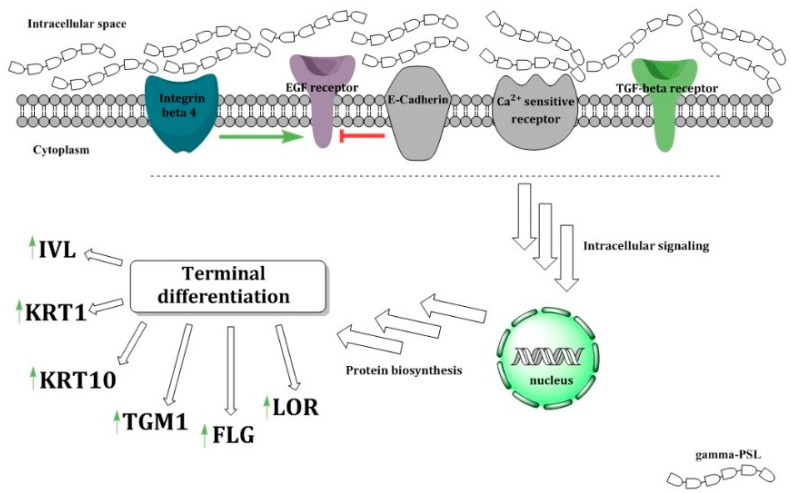
Potential mode of action of γ-PSL on NHEK, by interfering with the interaction of integrins on the cell surface with proliferation signals from the extracellular matrix.

As hypothesized, an interaction of γ-PSL with integrins or cadherins could explain the observed effects. Integrins are membrane-associated proteins. As long as they are connected to the extracellular matrix, they provide a constant proliferation signal mediated by interaction with the epidermal growth factor receptor. It might be possible that γ-PSL blocks this interaction of integrins with the extracellular matrix. On the other hand, cadherins initiate the differentiation process via cell contacts. γ-PSL could act as an artificial cell contact and feign the effect of the contact inhibition (Figure 6). The significant influence on tumor growth factor receptor TGFR after treatment of the cells with γ-PSL is observed (Figure 5) and it is hypothesized that the sulfonated, strongly negatively charged γ-PSL binds un-specifically to the cell surface, which again could influence the activity of the different receptor systems. It seems interesting that the mixed-linked β-1,3/1,4-linked glucans seem to interact more on the TGFB pathway towards terminal differentiation [10], while in contrast, the cellulose-like β-1,4-glucans act via EGFR-signaling [8] ]. It can be assumed that the differentiation-inducing effect of the glucans with 1,3-glucose residues in the backbone is due to an interaction with Dectin-1 on the surface of keratinocytes [27]. Again, this activates a cellular response of NHEK to the respective β-glucan [27]; this response might be similar to those described for 1,3/1,6-glucan from *S. cerevisiae*, which is defined as a pathogen-associated molecular pattern (PAMP) and a main structural cell wall compound in many fungi [27]. As β-1,4-glucans have not been recognized as PAMP-like polysaccharides, this can explain the different signaling pathways induced by the different polymers. In principle, it can be hypothesized that the differentiation-inducing effect of γ-PSL can be assessed as a response of the keratinocytes against β-1,3-glucans, mimicking a fungal attack. Increased keratinization of the cells could therefore reduce the destructive power of a fungal invasion.

From the aspect of the clinical use of γ-PSL and related glucans, it remains unclear what effects this increased keratinization will exert during the process of wound healing. Clinical studies are needed to explore the significance of this finding.

## 3. Materials and Methods

### 3.1. General Methodology

If not stated otherwise, all chemicals were purchased from VWR (Darmstadt, Germany). Lichenan was obtained from Sigma-Aldrich (Deisenhofen, Germany).

The following antibodies were used: β-actin (AC-15), mouse monoclonal (1:4000, 1 h at RT) from Sigma-Aldrich; KRT1 (1:200,000 for 2 h at RT), KRT10 (1:10,000 at 4 °C over night) and IVL (1:500,000 at 4 °C at RT), and rabbit monoclonals from Abcam; Rabbit Anti-Mouse IgG HRP conjugate from Jackson ImmunoResearch; and Rabbit anti-Mouse IgG HRP (1:10,000 for 45 min at RT) from Jackson Immuno Research. 

NMR spectra were recorded on an Agilent VNMRS 600 (Agilent Technologies, CA, USA). ^1^H and ^13^C-NMR measurements were obtained at 80 °C at 600 MHz and 150 MHz, respectively. ^1^H-^13^C-HSQC-NMR spectra were recorded at 20 °C. Data analysis was achieved with MestRneNova software version 10.0.0-14381. Sample concentration: 5 mg/mL D_2_O (Uvasol^®^, 99.8%, Merck, Darmstadt, Germany) and DMSO-D6 was added for referencing (δ 3.330 ppm). 

Gel permeation chromatography was performed on a low pressure Sepharose^®^6 stationary phase (GE Healthcare, Freiburg, Germany) using standard dextrans (Sigma, Deisenhofen, Germany) for calibration. Each fraction was tested on the carbohydrate content accordingly [28].

### 3.2. Synthesis of PSL

#### Synthesis of PSL was performed as decribed in Reference [12].

One gram of lichen (Sigma-Aldrich, St. Louis, MO, USA.) was suspended in 4 mL of NaOH solution (19 M). An amount of 13.8 mL of propan sulton (Sigma-Aldrich, St Louis, MO, USA) was added dropwise in 6 portions of 2.3 mL, followed by intensive stirring. The pH was adjusted to 7.0 by the use of sulfuric acid (1 M). The precipitated polymer was centrifuged (3000× *g*, 10 min) and washed 3 times with methanol. The polymer was dissolved in water and dialyzed for 5 days with double distilled water (MWCO 3500 Da), followed by lyophilisation. Yield: 46.9% (*w*/*w*), related to the starting material. ^1^H-NMR (D_2_O, 600 MHz, 80 °C): δ 5.38-5.37 (d, H-1 [β-1,3-Glc]); δ 5.17–5.15 (2 × d, H1 [β-1,4-Glc]); δ 4.60-3.98 (H2-H7); δ 3.60 (sb, H9); and δ 2.66 (sb. H8).^13^C-NMR (D_2_O, 600 MHz, 80 °C): δ 103.05 (C1 [β-1,3-Glc]); δ 102.77 (C1 [β-1,4-Glc]); δ 85.00 (C3), δ 79.22, 79.12 (C4), δ 76.25, 76.20 (C5), δ 75.42, 75.40 (C3), δ 74.74, 74.72 (C5), δ 73.87, 73.58, 73.44 (C2), δ 68.70 (C4), δ 61.32 (C7), δ 60.80, 60.76 (C6), δ 48.53 (C9), and δ 27.56 (C8); and correlations in part based on Reference [29]. 

### 3.3. Cells and Cell Culture 

#### Experiments were performed as described in Refenence [8].

Primary keratinocytes (NHEK) from juvenile human foreskin were obtained from Lonza (Basel, Switzerland) (Neonatal Epidermal Keratinocyte Progenitors, pooled cells from 3 subjects). To ensure validity of the treatment experiments, different cell batches have been used. 

HaCaT keratinocytes (Human adult low Calcium high Temperature) are spontaneously immortalized keratinocytes, established and kindly provided by Prof. Dr. Fusenig from the German Cancer Research Center DKFZ.

Primary keratinocytes were cultivated in a KGM Gold Keratinocyte Basal Medium (Lonza, Basel, Switzerland) plus KGM Gold Bullet Kit (Lonza, Basel, Switzerland) at 5% CO_2_, 37 °C. HaCaT cells were maintained in D-MEM high glucose with glutamine, 10% FCS (Biochrome, Berlin, Germany), 1% NEAA (PAA, Austria), and 1% Pen/Strep (Biochrome, Buchs, Switzerland) at 8% CO_2_/37 °C. Adherent cells were cultured until 70–90% confluence. 

### 3.4. Assays for Cell Physiology

Cellular proliferation rate was determined by BrdU Incorporation ELISA [13]; mitochondrial activity resp. cell viability was monitored by MTT assay [14]; and an investigation of the influence of test compounds on cell differentiation was determined, as described by Reference [8]. Western blotting: semi-dry transfer system (BioRad, Hercules, CA, USA), protein transfer: 30 min/10 V, followed by blocking with dry milk powder (Oxoid, UK) for 1 h at RT. Blots were incubated with diluted primary antibodies for 2 h at 4 °C, washed 3× for 5 min in TBS-T buffer and incubated for 45 min with diluted HRP-conjugated secondary antibodies. After washing for 3× for 10 min in TBS-T buffer, visualization was performed by the addition of freshly prepared and enhanced chemiluminescence (ECL) reagent (Biorad). 

### 3.5. qPCR, Isolation, Quantitation, and Reverse Transcription of Total RNA

Cells were seeded in 6-well plates (7.5 × 10^5^ cells per well). After incubation with test solutions for specific time intervals, the total cellular RNA was isolated using the RNeasy Mini Kit (Qiagen, Hilden, Germany) according to the manufacturer’s instructions. RNA quality and concentration were determined using µCuvette G1.0 and the BioPhotometer plus (both Eppendorf). Transcription into cDNA was achieved by the use of Transcriptor First strand cDNA synthesis Kit (Roche, Basel, Switzerland) according to the manufacturer’s instructions.

qPCR was performed using TaqMan gene expression assays (Applied Biosystems, Foster City, CA, USA) containing gene specific primers and probes (Table 2), as well as the SensiMix II Probe Lo-ROX Kit (Bioline), and the CFX96 Touch Real-Time PCR detection system (Bio-Rad) according to the manufacturer’s instructions. UBC, PPIA and TBP served as control genes. Data were evaluated by means of the CFX Manager 3.0 software from Bio-Rad.

### 3.6. Statistics

Statistics were performed using Microsoft Office Excel 2013 resp. 2016 and GraphPad Prism (Version 3). Results are expressed as interquartile mean ± interquartile ranges. The unpaired Student’s t test was used for the comparison against the untreated control with *p* < 0.05 (*) and *p* < 0.01 (**).

## 4. Conclusion

We have described the preparation of a cold-water soluble semi-synthetic mixed-linked β-1,3/1,4-d-glucan, which stimulates human keratinocytes by a specific mechanism into the terminal differentiation. A potential use of this cytokeratin-inducing polysaccharide might be used for future development to improve wound healing. Further studies have to be conducted to evaluate the clinical relevance of such compounds for improved wound healing and as additives for adhesive wound dressings. 

## Figures and Tables

**Table 1:** Known functionality of selected genes from qPCR screening array, which is related to the differentiation of keratinocytes.

Gene	Protein	Function
CDKN1A/p21	*Cyclin-dependent kinase inhibitor 1*	Influence on differentiation → G1 cell cycle arrest; initiation of differentiation in NHEK [17]
CHUK/IKKA	*Inhibitor of nuclear factor kappaB kinase subunit alpha*	Regulation of EGFR/Ras signaling; early and terminal differentiation of keratinocytes; interaction with E-cadherin [18,19]
FLG	*Filaggrin*	Structural protein of *cornfied envelopes*; marker of terminal differentiation [3]
FOSL1/Fra-1	*Fos-related antigen 1*	Forms AP1 complex together with JunB → interaction with integrin β4; influencing keratinocytes differentiation [20]
GNAQ	*Guanin nucleotide-binding protein G(q) subunit alpha*	G(q) coupled protein of the family of heterotrimeric guanine nucleotide binding G proteins [21]
KRT1	*Keratin, type II cytoskeletal 1*	Differentiation specific marker protein in NHEK; structural element of cornified envelope [3]
KRT10	*Keratin, type I cytoskeletal 10*	Differentiation specific marker protein in NHEK; structural element of cornified envelope [3]
MAP2K1	*Dual specificity mitogen-activated protein kinase kinase 1*	Part of the MAP kinase family; signaling; mostly coupled to membrane associated receptors; influence on cell cycle, proliferation, cell survival [22,23]
SP1	*Transcription factor Sp1*	Transcription factor; influence on differentiation of keratinocytes; regulation of loricrin, involucrin [24,25,26]
TGFB1R	*Transforming growth factor receptor beta* 1	Membrane associated receptor, Ser/Thr protein kinase type; forms heterodimeric complex with ligand TGFB1; regulates cell cycle, differentiation, proliferation, wound healing, formation of extra cellular matrix, immune suppression; further signaling via SMAD 2, 3, 4

**Table 2:** TaqMan gene expression assays used for gene expression studies of NHEK by qPCR.

Gene	Assay-ID	Gene	Assay-ID
PPIA	Hs04194521_s1	IVL	Hs00846307_s1
UBC	Hs00824723_m1	TGM1	Hs00165929_m1
TBP	Hs00427621_m1	SMAD2	Hs00183425_01
KRT1	Hs00196158_m1	SMAD3	Hs00706299_s1
KRT10	Hs00166289_m1	TGFB1	Hs00998133_m1
LOR	Hs01894962_s1	TGFB1R	Hs00610320_m1
FLG	Hs00856927_g1	TGM1	Hs00165929_m1

## Supplementary Materials

The following are available online.

## Abbreviations

CK	cytokeratin
DP	degree of substitution
EGFR	epidermal growth factor receptor
FLG	filaggrin
HaCaT	Human adult low Calcium high Temperature keratinocyte cell line
KRT	(cyto)keratin
IVL	involucrin
LOR	loricrin
NHEK	primary normal human epidermal keratinocytes
PSL	γ-propoxy-sulfo-lichenan
qPCR	quantitative real-time polymerase chain reaction
TGM	transglutaminase
UC	untreated control
