# Supplementary material for: γ-Propoxy-Sulfo-Lichenan Induces In Vitro Cell Differentiation of Human Keratinocytes
Source: Molecules. 2019 Feb 5;24(3):574. doi: 10.3390/molecules24030574 (PMC6384931; doi:10.3390/molecules24030574)

Supplementary Data

γ-Propoxy-sulfo-lichenan induces *in vitro* cell differentiation of human keratinocytes

Stefan Esch [1], Maren Gottesmann [1] and Andreas Hensel [1,*]

[1] University of Münster, Institute of Pharmaceutical Biology and Phytochemistry, Corrensstrasse 48, D-48149 Münster, Germany

[*] Correspondence: ahensel@uni-muenster.de; Tel.: +49-251-8333380; Fax: +49-251-8338341

Figure S1: Gel permeation chromatography elution profile of γ-propoxy-sulfo lichenan (γ-PSL) on Superose™6, eluted with NaCl 0.1 mol/L. Calibration has been performed with standard dextrans.

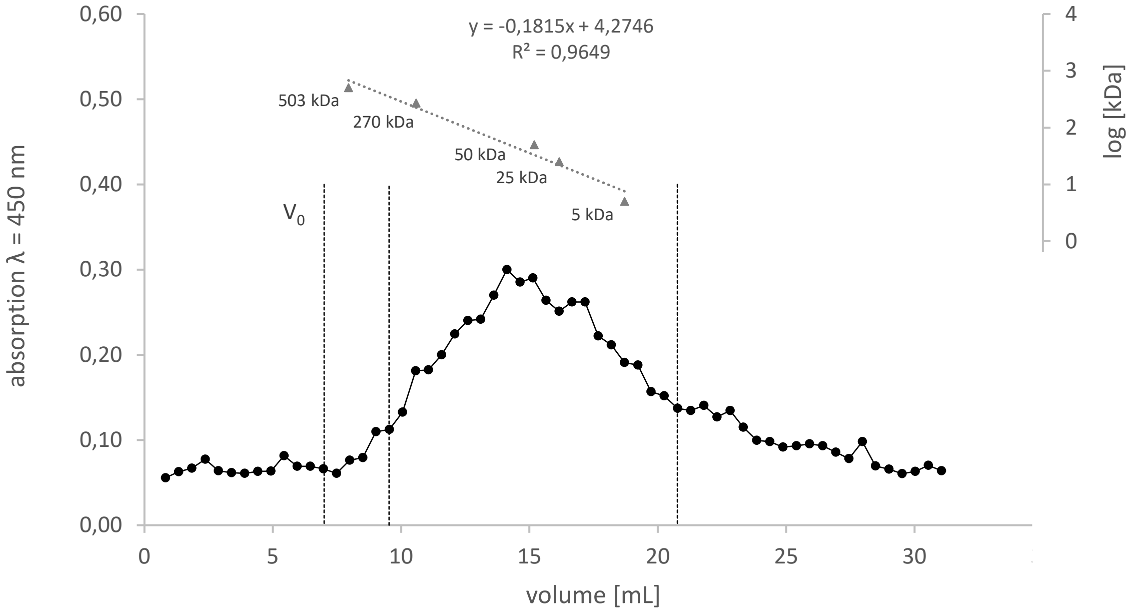

Figure S2: Influence of γ-PSL on the relative cell viability (48 h, MTT assay) (**A**) and on cell proliferation (24 h, BrdU incorporation ELISA) of NHEK (**B**). Data are related to the untreated control (UC). PC: FCS 5%. *: $p < 0.1$, **: $p < 0.05$*: $p < 0.01$.

A

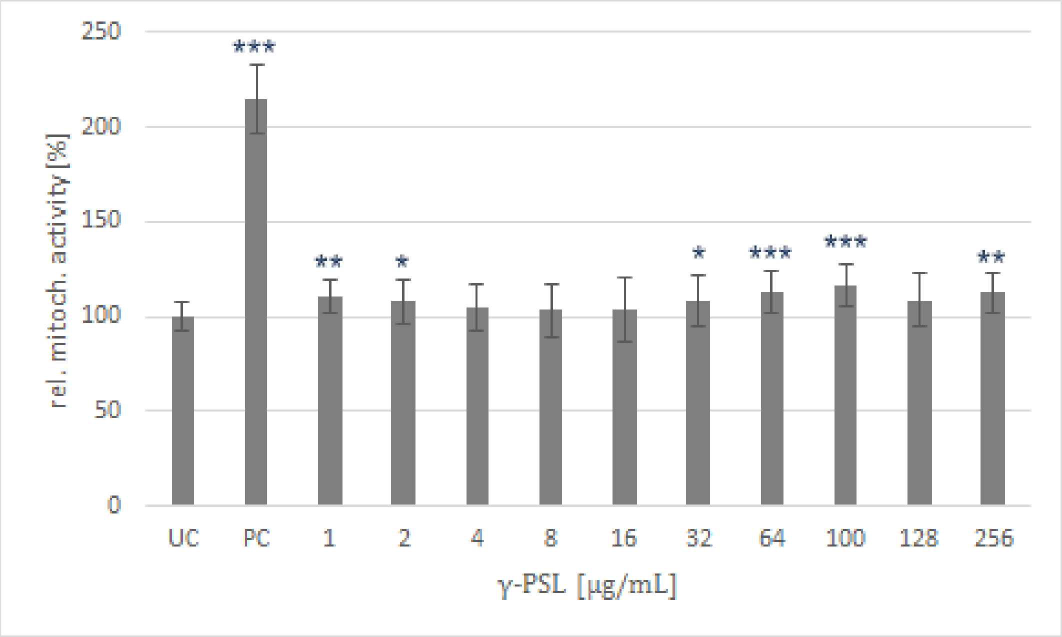

B

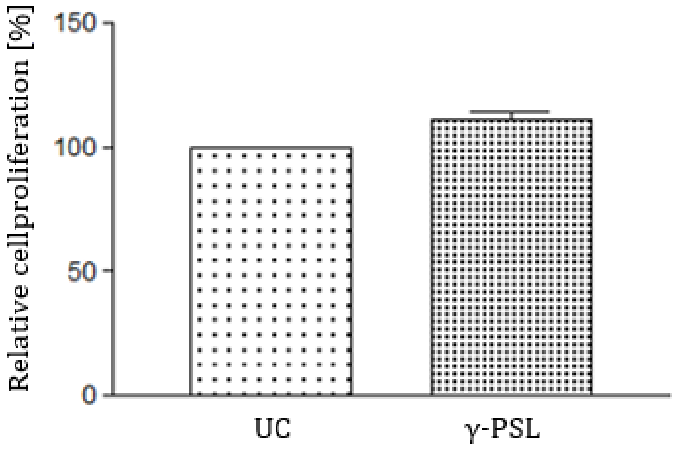

Supplement: Supplementary file 1 [file molecules-24-00574-s001.pdf]
